# Direct observation of molecular arrays in the organized smooth endoplasmic reticulum

**DOI:** 10.1186/1471-2121-10-59

**Published:** 2009-08-24

**Authors:** Vladimir M Korkhov, Benoît Zuber

**Affiliations:** 1 MRC Laboratory of Molecular Biology, Hills Road, CB2 0QH, Cambridge, UK; 2 Institute of Molecular Biology and Biophysics, ETH Hoenggerberg, Schafmattstr. 20, 8093 Zurich, Switzerland

## Abstract

**Background:**

Tubules and sheets of endoplasmic reticulum perform different functions and undergo inter-conversion during different stages of the cell cycle. Tubules are stabilized by curvature inducing resident proteins, but little is known about the mechanisms of endoplasmic reticulum sheet stabilization. Tethering of endoplasmic reticulum membranes to the cytoskeleton or to each other has been proposed as a plausible way of sheet stabilization.

**Results:**

Here, using fluorescence microscopy we show that the previously proposed mechanisms, such as membrane tethering via GFP-dimerization or coiled coil protein aggregation - do not explain the formation of the calnexin-induced organized smooth endoplasmic reticulum membrane stacks. We also show that the LINC complex proteins known to serve a tethering function in the nuclear envelope are excluded from endoplasmic reticulum stacks. Finally, using cryo-electron microscopy of vitreous sections methodology that preserves cellular architecture in a hydrated, native-like state, we show that the sheet stacks are highly regular and may contain ordered arrays of macromolecular complexes. Some of these complexes decorate the cytosolic surface of the membranes, whereas others appear to span the width of the cytosolic or luminal space between the stacked sheets.

**Conclusion:**

Our results provide evidence in favour of the hypothesis of endoplasmic reticulum sheet stabilization by intermembrane tethering.

## Background

A key function of most cellular membranes is to form organelles enclosing biochemically distinct subcompartments in the cell essential for a multitude of cellular processes. The endoplasmic reticulum (ER) comprises the most abundant and highly versatile component of the endomembrane system. The specialized sub-compartments of the ER include: (i) the nuclear envelope (NE), composed of two adjacent membrane sheets surrounding the nucleus, and (ii) the peripheral ER, including membrane sheets and a complex network of tubules [1,2]. ER has traditionally been classified into rough and smooth, based on early electron microscopy observations, where the rough ER was distinguished by the presence of ribosomes on its surface, and the smooth one by their absence [3].

The overall architecture of the ER is highly evolutionarily conserved from yeast to mammals, with luminal intermembrane distances ranging between 50 and 100 nm [4]. Inter-conversion between tubes and sheets of ER has been proposed based on their varying abundance during various stages of cell cycle [5]. This clearly indicated that active components are involved in shaping the ER. In line with this suggestion, proteins such as the reticulons and DP1, that induce high membrane curvature and thus stabilize ER tubes, have recently been identified [6,7].

In contrast, the mechanism of ER sheet stabilization has been elusive and the identities of the proteins involved are unknown. Several protein complexes have been proposed to stabilize and sustain the extended flat double sheet morphology of the nuclear envelope, such as the SUN proteins that span the whole width of the NE lumen, connecting the nucleus to the cytoskeleton via Nesprin family proteins [8]. The peripheral ER sheets may be stabilized by tethering to the cytoskeleton by, for example, Climp63, which is a microtubule-binding protein [1,4,9]. Weak interactions between fluorescent protein tags engineered onto ER-resident proteins, such as cytochrome b(5) or Sec61, have been proposed to stabilize ER sheet morphology and to induce formation of organized smooth ER, OSER [10,11]. A range of morphologies, including cubic [10,12], tubular [13] and stacked sheet OSER have been identified, based on electron microscopy. Because of the highly ordered arrangement of these large membranous assemblies, it has been suggested that OSER may serve as a paradigm for membrane and organelle biogenesis at molecular level [14].

While working on interactions of ER chaperones with neurotransmitter transporters, we found that overexpression of calnexin, an ER-resident lectin chaperone with a single transmembrane-spanning domain, induces formation of stacked OSER membranes, detected by fluorescence and cryo-electron microscopy [15]. We have also detected OSER membranes in untransfected mammalian cells, by immunocytochemical labelling of endogenous calnexin [15]. These structures are highly dynamic and contain mobile, non-aggregated membrane protein pools [10,11,15]. OSER-like membrane structures have been observed previously in certain pathological conditions in vivo, e.g., in Emery-Dreifuss disease, torsion dystonia and Hodgkin's lymphoma [16-18]. Thus, OSER membrane expansion in eukaryotic cells may represent a physiologically relevant response of cells to stress, i.e., excessive production of misfolded or misassembled proteins. Such response mechanistically resembles the ER stress-induced ER expansion in yeast, where sequestration of the ER membranes into autophagosome-like multilamellar structures, but not their autophagic degradation, is essential for survival [19]. This suggestion is corroborated by the observation that OSER structures induced in mammalian cells are not subject to bulk degradation via lysosomes/autophagosomes [20].

Here, we revisit the mechanisms of ER sheet stabilization and stacking and present a detailed investigation into the organization of the stacked smooth ER sheets. Our results indicate that the OSER membrane stacks are highly regular structures that may be maintained through the ordered tethering of membranes by a large native complex, rather than through interactions between heterologously overexpressed proteins.

## Methods

### Reagents, and cell lines and DNA constructs

DMEM and standard cell-culture reagents were from GIBCO (Paisley, UK). SUN1 and SUN2 antibodies were a generous gift from Sue Shackleton (Leicester, UK); Nesprin-1 antibody was from Abcam (Cambridge, UK). Anti-rabbit antibody conjugated with Texas Red was from Invitrogen (Paisley, UK). Climp63-GFP construct was generously provided by Hans-Peter Hauri (Basel, Switzerland). Calnexin constructs were described previously [15].

### Cell culture

HEK293 cells were cultured at 37°C, supplemented with 5% CO_2_. Transfections were performed using Lipofectamine-2000 (Invitrogen), according to supplier's instructions. A total of 2-4 μg of plasmid DNA were used in an individual transfection reaction per 3 cm plate; 10 μg of plasmid DNA were used to transfect cells growing in 10 cm dishes.

### Confocal microscopy

For confocal microscopy cells were grown on poly-L-glutamine-coated (Sigma) glass cover-slips until 50-80% confluence, followed by transfection and/or fixation. Cells were fixed with 4% paraformaldehyde (Sigma) in phosphate-buffered saline (PBS), permeabilized for 30 min at room temperature using PBS with 1% BSA and 0.01% Triton-X100 and stained for 1 h with primary and secondary antibodies (in PBS, 1% BSA) where appropriate. Coverslips with stained cells were washed four times in PBS and mounted onto glass slides in Vectashield medium (Vector Laboratories) for microscopy. Images were acquired using Zeiss LSM 510 confocal microscope with a 63× objective lens.

### Cryo-electron microscopy of vitreous sections

The procedure of cell preparation for cryo-electron microscopy of vitreous sections (CEMOVIS) was described previously [15]. Briefly, cells were centrifuged for 5 min at 1400 rpm and resuspended in 30% dextran-PBS (average mass of dextran - 40 kDA; Sigma-Aldrich, Buchs, Switzerland). The cells were introduced into the 200-μm deep cavity of a copper membrane carrier (Leica, Vienna, Austria) and vitrified by high pressure freezing with a Leica EMPACT2 apparatus. Membrane carriers were clamped in the specimen holder of an FC6/UC6 cryo-ultramicrotome (Leica) and trimmed in pyramidal shape. Copper was trimmed away with a diamond knife (Diatome, Bienne, Switzerland) on part of the specimen holder and the specimen was trimmed to pyramidal shape with the same knife. 50-nm feed cryosections were cut with 35° diamond knife (Diatome) under standard cutting conditions, at -140°C. The cryosections were collected on 1000-mesh grids (Agar Scientific, Essex, United Kingdom) coated with carbon and stored in liquid nitrogen or transferred immediately to the microscope. The images were acquired with Tecnai T12 microscope (FEI Company, Eindhoven, Netherlands) on film, under standard low-dose imaging conditions, using 26,000-30,000× magnification, at -1.5 to -2.5 μm defocus and 120 kV.

Images were scanned and digitised using an MRC KZA scanner at pixel size of 2 or 2.3 Å, dependent on magnification during cryo-EM experiments. All image analysis - selection of the regions of interest, distance measurements, Fourier transformation, identification of strong reflections - was performed using Ximdisp [21]. For measurement of the intermembrane distances, the micrographs showing OSER membrane stacked parallel (13 micrographs) and perpendicular (4 micrographs) to the cutting direction, respectively. The distances between the middle points of the juxtaposed membrane sheets were measured in the areas of interest where the stacked membranes were parallel to each other. The distance measurements in stacks perpendicular to the cutting direction were unaffected by specimen compression during the cutting procedure; the comparisons of the parallel vs perpendicular cytosolic distances, which showed relatively small S.D. values, revealed an approximately 30% compression of the specimen due to cutting. MRC Image2000 software programs TRMASK and FFTRANS [22] were used for masking and Fourier transformation/filtered image calculation, respectively. Briefly, well-preserved regions of interest were boxed and rotated to align the membranes vertically. Fourier transforms were calculated and strong reflections, including the equatorial data, were masked. To visualize the features of interest, the filtered images were calculated using the masked Fourier transforms.

Inspection of the images confirmed that appearance of strong reflections in the selected regions of interest does not correlate with the direction of the knife marks (data not shown). The artefacts caused by the diamond knife - knife marks and crevasses (normally perpendicular to each other) - in most cases result in appearance of very low resolution reflections (250-350 Å), outside the range where we observed ordered features (40-100 Å). Image analysis was performed on images devoid of crevasses.

## Results

### Fluorescent protein tag oligomerization does not cause ER stacking

We have previously shown that calnexin-YFP or calnexin-CFP overexpression causes OSER expansion, based on immunofluorescence and low-magnification CEMOVIS images [15]; we also observed OSER-like inclusion in non-transfected mammalian cells by staining for endogenous calnexin [15]. In contrast, the findings of Snapp et al. (2003) suggested that GFP-tagged membrane proteins residing in the proximal ER membranes could weakly interact with each other, causing the membranes to stack [10]. This stacking was proposed to be mediated either via interactions between the intrinsic extramembranous domains of the ER proteins, or by self-association of the fluorescent protein tags. OSER induction in the case of GFP-tagged proteins was abolished when non-oligomerizing GFP mutants were used [10].

**Figure 1:**
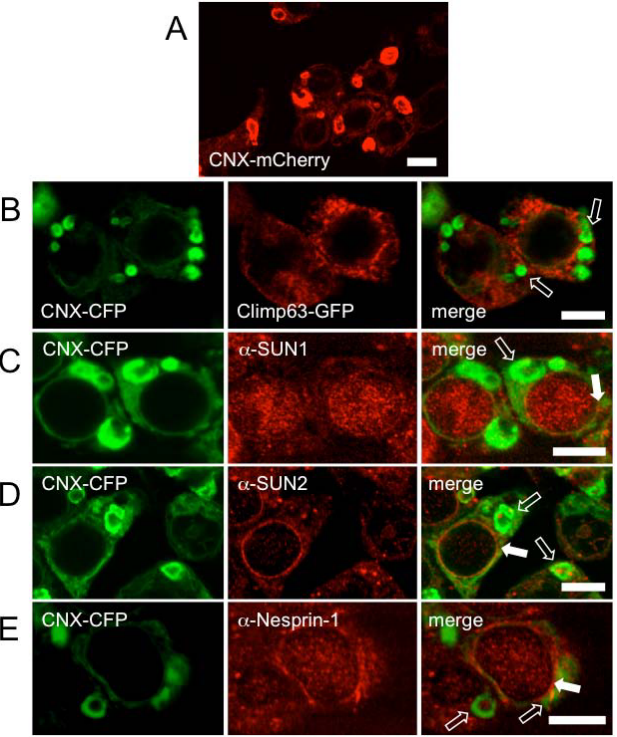
**OSER membrane biogenesis is sustained by monomeric fluorescent protein fusion expression and does not involve Climp63 and SUN proteins**. *A*. HEK293 cells grown on coverslips were transfected with calnexin-mCherry (CNX-mCherry) construct and observed by confocal microscopy 24 hours later, as described under "Materials and methods". OSER membrane expansion was observed in almost every expressing cell (n = 14). *B*. HEK293 cells were co-transfected with calnexin-CFP (CNX-CFP) and Climp63-GFP constructs and processed as in *A*. OSER structures positive for calnexin and negative for Climp63-GFP are indicated by an arrow (number of cells showing Climp63-GFP excluded from the OSER membranes, n = 23). *C-E*. HEK293 cells grown on glass coverslips transfected with calnexin-CFP were cultured for 24 h and labelled with an antibody for endogenous SUN1 (*C*), SUN2 (*D*) or Nesprin-1 (*E*), stained with a Texas Red-conjugated secondary antibody and mounted on glass slides for confocal microscopy. All but one cell stained for SUN1 (n = 56) or SUN2 (n = 35), showed exclusion of the co-stained SUN proteins from the OSER; the calnexin/SUN colocalization observed in a single cell of each set was presumably due to fortuitous targeting of SUN proteins into the OSER structures (some of which may originate from the NE [10]). All Nesprin-1-stained cells cells showed its exclusion from the OSER (n = 34). Multilamellar OSER structures positive for calnexin-CFP but negative for the co-stained proteins are indicated by open arrows; solid arrows indicate SUN1-, SUN2- or Nesprin-1-stained NE (SUN1 and Nesprin-1 labelling produced intra-nuclear staining, in addition to the NE staining, under various experimental conditions). Scale bar in each image is 10 μm.

We therefore tested whether calnexin tagged with a monomeric fluorescent protein would be capable of inducing multilamellar ER stacks. If fluorescent protein dimerization were a prerequisite for OSER formation, one would expect that calnexin tagged with a monomeric fluorescent protein should not induce multilamellar bodies. As shown in Figure 1A, this was not the case - calnexin tagged with monomeric Cherry fluorescent protein [23] was as potent in OSER induction as calnexin tagged with enhanced YFP or CFP [15]. This suggested that fluorescent protein moiety of the calnexin-mCherry fusion protein is unlikely to induce OSER formation in cells that overexpress it.

### Climp63 is excluded from OSER

Another hypothesis of ER sheet stabilization and stacking involved Climp63, a microtubule-binding ER protein [9]. Climp63 contains an extended luminal coiled-coil domain, and upon purification forms large rod-shaped aggregates. These structures appear sufficiently long to span ER lumen, and thus could be the prime candidates for ER sheet stabilization and stacking.

We co-expressed calnexin-CFP with Climp63-GFP in HEK293 cells. No colocalization of the two proteins in the OSER could be observed (Figure 1B); Climp63-GFP was completely excluded from the calnexin-positive multilamellar bodies. This suggested that Climp63-GFP does not participate in stabilization of the OSER stacks.

### LINC complex proteins are not involved in OSER stacking

Some of the OSER compartments were previously suggested to be derived from NE membranes [10,12], making the involvement of the NE-stabilizing proteins in OSER plausible. Proteins of the NE that participate in the so-called LINC complex, SUN1 and SUN2, span the entire width of the NE connecting NE inner membrane with NE outer membrane [8]. High levels of over-expressed ER proteins essential for production, folding and assembly of membrane proteins could perturb the normal biogenesis of SUN proteins leading to their 'leakage' into the peripheral ER instead of the NE, inducing ER membrane stacking. Thus, a possible explanation for OSER induction by ER protein overexpression could be their effect on targeting of SUN proteins.

To test this hypothesis, we used immuno-fluorescence to visualize the endogenous SUN proteins in cells overexpressing calnexin. Antibody staining of SUN1 and SUN2 revealed the presence of SUN proteins in the NE, partially colocalizing with calnexin-CFP (Figure 1C and 1D). However, similarly to Climp63-GFP, endogenous SUN proteins were excluded from the calnexin-induced OSER structures.

We have also tested whether endogenous Nesprin-1 enters the multilamellar bodies. Proteins of the Nesprin family include several giant isoforms (M.W. up to 1 MDa) with large cytosolic regions. The classical function of Nesprins is to link the nuclear envelope to actin cytoskeleton [8,24]. Cytosolic regions of Nesprins contain spectrin repeats that may undergo oligomerization. Thus, Nesprins could fulfil the criteria for tethering of ER membranes and inducing the OSER stacks. However, similarly to SUN proteins, we found Nesprin-1 to be excluded from calnexin-CFP stained OSER structures (Figure 1E). Based on these experiments, involvement of LINC complex-participating proteins (SUN1-2 and Nesprin-1) in OSER biogenesis was ruled out.

### Cryo-electron microscopy of vitreous sections reveals periodicity of OSER membrane packing

To obtain a more detailed view of the ER membrane stack organization, we performed a CEMOVIS analysis of HEK293 cells over-expressing YFP-tagged calnexin. CEMOVIS is a method that preserves the native structure of the cell and allows imaging of the cellular architecture at the molecular level [25]. We have used this method previously to show that calnexin-induced OSER structures are indeed multilamellar [15].

**Figure 2:**
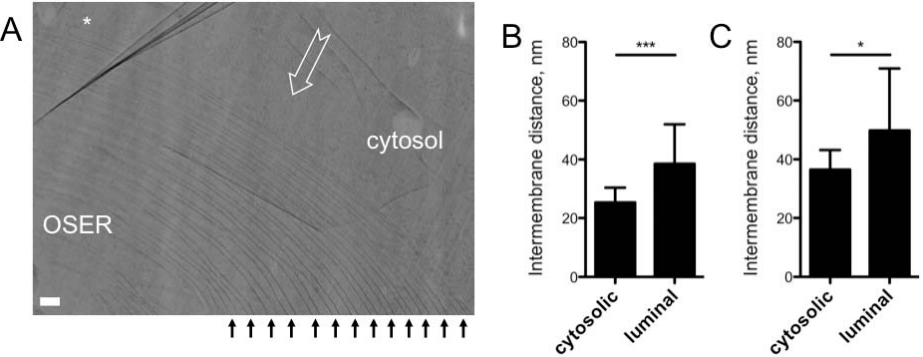
**CEMOVIS reveals regular cytosolic intermembrane distances within OSER stacks**. *A*. A typical projection view of a 50 nm-thick vitreous section of the OSER shows a regular spacing between OSER membranes on their cytosolic side (indicated by black arrowheads). HEK293 cells transfected with calnexin-YFP construct were processed for CEMOVIS analysis, as described in detail under "Materials and methods". Standard low-dose imaging conditions with 26,000-30,000× magnification were used to perform cryo-EM experiments; images were acquired on film. Open arrow indicates the cutting direction; asterisk indicates the fold of the peripheral OSER membrane. Scale bar corresponds to 100 nm. *B*. Intermembrane distances on cytosolic and luminal sides of OSER membranes were sampled from 13 micrographs in which membrane stacks were aligned along the cutting direction, as described in "Materials and Methods"; n values are 86 and 81 for cytosolic and luminal intermembrane distances, respectively; asterisks indicate P < 0.0001. *C*. As in B, but the distances were measured from 4 micrographs in which the membrane stacks were aligned perpendicular to the cutting direction; n = 17 for cytosolic and luminal distances. Asterisks indicate P < 0.05. Data in *C-D *are shown as mean ± S.D.

Here, we analysed a total of 55 good-quality vitreous section micrographs. The high magnification CEMOVIS imaging revealed the absence of a delimiting membrane (Figure 2A shows a peripheral OSER membrane sheet fold, marked with an asterisk), consistent with the OSER structures being continuous with the rest of ER [11]. The most striking feature of the OSER structures was the uniform intermembrane spacing of the cytosolic and luminal compartments (Figure 2A; cytosolic space within OSER structure indicated by black arrowheads). Measurements in the OSER stacks aligned along the cutting direction revealed the average distances of 25.3 nm between the cytosolic, and 38.6 nm between the luminal OSER membrane faces (Figure 2B). The same measurements performed on membranes aligned perpendicular to the knife-marks, with the intermembrane distances measured perpendicular to the cutting direction and thus unaffected by sample compression, revealed the average distances of 36.5 nm and 49.8 nm for cytosolic and luminal sides, respectively (Figure 2C). Based on the comparisons between the cytosolic distances measured parallel and perpendicular to the cutting direction, we estimated a compression rate of about 30%, as typically observed in CEMOVIS experiments. The highly significant difference between the distances in the two compartments, consistent in both the compressed and the non-compressed dimension, provided the first indication of an active component pertaining to the stacking of OSER membranes.

The long distances between cytosolic and luminal faces of the membranes clearly indicated that overexpressed proteins on their own would not be able to stabilize the OSER. Calnexin, even tagged with a fluorescent protein (the length of the YFP barrel is ~4 nm) on its cytosolic C-terminus (90 amino acid residues), would not be able to reach out and connect two ER membranes. Likewise, the length of the luminal domain of calnexin, based on its X-ray structure [26], is insufficient to bridge the juxtaposed OSER membranes.

### CEMOVIS reveals macromolecular arrays within OSER

**Figure 3:**
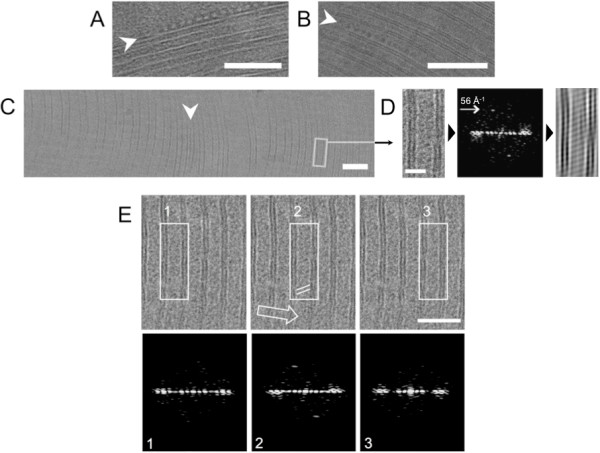
**CEMOVIS reveals the presence of molecular arrays at the OSER membranes**. *A-B*. OSER membranes from HEK293 cells transfected with calnexin-YFP were imaged by CEMOVIS as described in the legend for Figure 2. Large globular proteins/protein complexes were found arrayed at the surface of the delimiting membrane (*A*) and in the cytosolic space between the OSER membranes (*B*; arrays are indicated by arrowheads). Scale bar is 100 nm. *C*. Regions of interest within an OSER stack, encompassing two membranes. Arrowhead indicates presence of large complexes, as in *A *and *B*; scale bar is 100 nm. *D*. The boxed areas were rotated to align the membranes vertically, their Fourier transforms were calculated. The Fourier transforms were inspected for presence of strong reflections that would be indicative of ordered arrangement of the densities within the micrograph; the reflection in *D *corresponds to striations with a period of 56 Å^-1^. The masked transforms were used to produce a filtered image, visualizing the ordered features (*D*, rightmost panel). *E*. Ordered densities are confined to the individual intermembrane regions of interest. The indicated regions of interest were processed as described under "Materials and methods". Of the three adjacent regions, only region "2" shows a reflection in the Fourier transform. Open arrow indicates the cutting direction; white lines in the boxed area indicate the orientation of the ordered density between the membranes within region "2".

The ectopically expressed membrane proteins inducing and/or populating the OSER membranes have previously been shown to be mobile, rapidly diffusing in the bilayer plane [10,15]. FRET experiments using calnexin-CFP/calnexin-YFP constructs suggested that proteins are packed tighter in the OSER membranes than in the rest of peripheral ER [15]. Consistent with these findings, some of the CEMOVIS micrographs showed closely spaced arrays of globular complexes at the cytosolic face of the OSER membranes, either at the outmost membrane of the multilamellar body (Figure 3A), or trapped within it (Figure 3B). The lengths of observed areas displaying such arrays were in the range of 100-200 nm. The diameter of individual particles was about 10-12 nm, about half the size of a ribosome. The identity of the complexes is therefore not known. However, this observation is the first direct observation of arrayed macromolecular complexes in the OSER membranes of the mammalian cells preserved in a hydrated native-like state. Because of the nature of the technique and the limited thickness of the specimen (50 nm) it was only possible to obtain a 1-dimensional projection view of these arrays, perpendicular to the membrane plane. It is possible that these arrays extend in two dimensions on the cytosolic surface of OSER membrane.

### Molecular arrays tethering stacked ER sheets

The high regularity of the ER membrane stacks suggested that tethering complexes could exist. A mechanism similar to that by which coiled-coil proteins organize Golgi membrane stacks [4] could account for ER sheet stabilization and stacking. We set out to perform a more in-depth analysis of our CEMOVIS images to detect the presence of molecular tethers between adjacent stacked membranes.

The images obtained by CEMOVIS are very noisy, allowing only the gross features, such as membranes or large protein complexes (e.g., ribosomes and microtubules), to be observed directly. Visualization of smaller objects directly is, in most instances, not possible. However, if the molecules are arranged in an orderly fashion, this can be detected in Fourier space and, if the order is sufficiently good, the structures of the arrayed molecules can be determined to near atomic resolution in favourable cases, e.g., from 2D crystals of membrane proteins [22]. We therefore analyzed the images in Fourier space, as described below.

The image analysis routine included digitization of the micrographs and selection of well-preserved regions of interest (as shown in Figure 3C). We excluded micrograph areas in which the tilt angle of the membranes relative to the section plane was strongly deviating from 90°, as well as the areas where the specimen was severely damaged by freezing and cryo-sectioning procedures (e.g. where crystalline ice and/or crevasses were observed). Selected regions of interest included two membranes and an intermembrane space (either luminal or cytosolic). Fourier transforms of the boxed regions were calculated and inspected for the presence of strong reflections along or at an angle to the meridian (as in Figure 3D), which would be indicative of an ordered density pattern within the analysed area.

To exclude the possibility of an artefact caused by sample preparation or imaging, 3 adjacent areas of the same image were analysed as described above (Figure 3E). Only the middle region of interest (region "2") showed a reflection at 56 Å^-1^, whereas the regions on either side of region "2" showed no ordered densities above the noise level. This indicated that the features detected in region "2" are not extending over a large area of the micrograph, but are confined to the region of interest between the membranes. The same image also illustrates a distinction between orientation of the striations between the membranes (indicated by white lines within the boxed region "2") and the cutting direction (indicated by an open arrow). We found no correlation between the cutting direction and the observed ordered densities in the best images, suggesting that these features are not created during sample preparation (data not shown).

**Figure 4:**
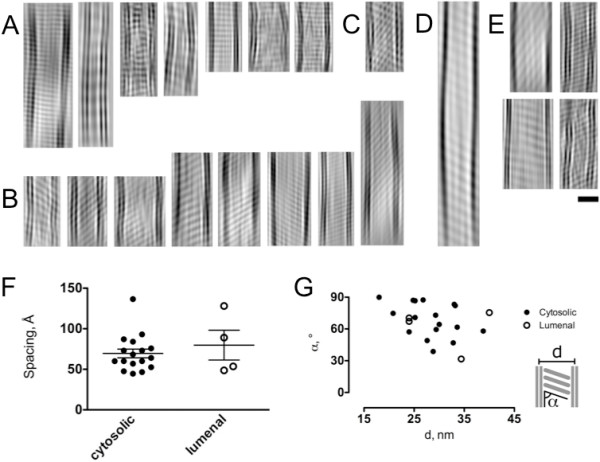
**Comparison of the ordered densities within the OSER**. *A-D*. The images were generated using the information in the masked Fourier transforms of the ROIs in the digitized micrographs, as described in "Materials and methods" (panels *A-D *show the cytosolic regions, whereas *E *shows the luminal regions); scale bar in *E *corresponds to 20 nm; all regions in *A-E *are scaled equally. The images with striation angles close to 90° are shown in *A*, whereas the regions with sharper striation angles are shown in *B*. An area in which two separate sets of striation overall producing a composite pattern is shown in C. An extended cytosolic region shows broadening of the spacing between the memrbanes, flanked by ordered densities (*D*). *F-G*. Geometric parameters of the ordered densities between OSER membranes indicate high degree of flexibility. *F*. The spacings between the striations (corresponding to the position of the strong reflection in the Fourier transform) were plotted for the identified cytosolic (black circles) and luminal (white circles) regions of interest; mean ± S.D. are shown in the graph. *G*. The distances between OSER membranes (d) were plotted against the angles between striations and membranes (α); an inset sketch indicates depicts the meaning of "d" and "α". Data points corresponding to the cytosolic and luminal regions of interest are indicated by solid and open circles, respectively.

Analysis of the selected regions of interest revealed the presence of periodic densities, indicative of the ordered arrangement of molecules between the OSER membranes. Unfortunately, several factors forbid analysis of the vitreous sections of OSER membranes as 2D crystals: (a) the vitreous sections of OSER membranes represent only slices across the tentative 2D crystalline areas; (b) as a soft biological material, the OSER membranes are prone to distortions, bending and folding; (c) even if the specimen is crystalline, minute changes in the specimen thickness may lead to data corruption due to inclusion of disordered regions or extraneous ordered regions in the final projection. Together, these factors prohibit indexing of the observed diffraction patterns. Therefore, for illustration purposes, using only the strong reflections we masked the Fourier transforms imposing a simple 1-dimensional lattice and calculated filtered images of the regions of interest by back-transformation, visualizing the density features between the stacked ER membranes (Figure 4).

Based on our selection criteria, in a total of 55 micrographs, we found 17 reasonably well-ordered regions on the cytosolic side (Figure 4A-D) and 4 reasonably well-ordered regions of interest on the luminal side (Figure 4E) connecting two adjacent OSER membranes. The densities that were visualized between the OSER membranes were represented by the sets of parallel striations, at an angle to the two ER membranes. The period and angle of striations in the luminal and cytosolic regions were similar: although in some cases the striations were at an approximately 90° angle to the plane of the membrane (Figure 4A), in others the angle was sharper (Figures 4B-E; the angles observed in all images are plotted in Figure 4F). In several cases discontinuities in the ordered regions were observed, as in Figure 4C, where such feature coincides with an apparent broadening of the intermembrane distance. Although the sampling of the luminal regions of interest was limited (n = 4), the average observed luminal inter-striation spacing appeared similar to the cytosolic one (Figure EA). We found no correlation between the intermembrane distances and the striation/membrane incident angles (Figure 4F).

The observation of densely packed, periodic structures connecting two ER membranes suggests that these structures may act as molecular tethers. An absence of correlation between the parameters of these tethers, namely the spacing, the angle and the distance between the membranes, is indicative of high flexibility of these tethering complexes, which in turn is in line with the previously suggested dynamic nature of the proteinaceous matrix that shapes the ER [27].

## Discussion

To the best of our knowledge, this is the first report detailing ER membrane sheet organization in a native environment, i.e. unfixed, hydrated cells. To date, the reports of attempts to visualize the ultrastructure of the ER membranes included freeze fracture electron microscopy of the crystalloid ER (tubular OSER) in the UT-1 cells, which showed macromolecular complexes of approximately 10 nm in diameter [28], and a freeze fracture/deep etch electron microscopy analysis of endomembranes of various cells, which revealed existence of 'bridges' connecting the membranes of different organelles [29]. However, both reports had common problems inherent to the employed methodology: the electron microscopy was performed on the dehydrated and/or fixed specimen, after shadowing with platinum and carbon - the replicas were subjected to imaging. This implies that the observed structures could be artefacts, e.g. due to aggregation of material during the etching process, demeaning the value of those observations.

We used CEMOVIS methodology to preserve the native ultrastructure and to image the intracellular membranes of the HEK293 cells by cryo-EM at unprecedented level of detail. A sub-compartment of ER, OSER, was used as a model system, which allowed us to sample a sufficient amount of stacked ER sheet micrographs for analysis. Using projection images of the OSER membranes we have identified ordered cytosolic and luminal macromolecular arrays supporting ER membrane stacking. The imaging of the OSER membranes may not allow us to measure directly the interaction between OSER membranes and thus we may not unequivocally rule out the possibility that the ordering of the molecules is secondary to ER stack formation. However, we propose that the OSER membranes may be stabilized by these arrays.

We have shown that induction of ER sheet stacks by YFP-tagged calnexin overexpression is not driven by fluorescent protein dimerization. Monomeric fluorescent protein-tagged calnexin induces OSER formation; the stacked membranes are too far apart for direct *in trans *interaction between overexpessed proteins. It is unlikely that the discrepancy between our results and those published previously [10] resulted from usage of different cell types, cell culture conditions, reagents and DNA constructs. Mammalian cells were used throughout and similar vectors were used for transfections in both studies; the proteins employed to induce OSER (cytochrome b(5), Sec61[10] and calnexin [15]) are known to exist in a complex with each other [30], and are therefore likely to reside in the same compartments. In addition, OSER-like structures have been observed in mammalian cells in the absence of any protein overexpression, with or without GFP involved [15]. It is possible that under certain circumstances, depending on the structure of a particular fusion construct, the presence of GFP may indeed be conducive to *trans*-interaction between the ER membranes leading to membrane tethering. It is worth noting that a broad range of ER-localized proteins have been known to induce OSER formation, including: cytochrome b5 [31], HMG-CoA reductase [28], inositol 1,4,5-trisphosphate receptor [32], cytochrome P-450 [33], microsomal aldehyde dehydrogenase [34], torsinA [17] and others. Therefore, it can be anticipated that more than one element in the structure of these very different proteins should affect their ability to cause rearrangements in the ER ultrastructure.

Several intracellular compartments, both in prokaryotes and eukaryotes, have been studied by CEMOVIS previously with a comparable level of detail [35-41]. Ordered arrangement of trans-cleft molecular complexes in the neuronal synapses has been visualized using this technique [42]. Recently published tomographic reconstruction of desmosomal vitreous sections revealed quasi-crystalline arrays of cadherin molecules connecting the skin cells [25,43]. We propose a model whereby OSER membranes are stabilized in a similar way, i.e., adjacent ER membranes are connected by extended arrays of intracellular 'adhesion' molecules, although our results indicate that the OSER-stabilizing structures are more disordered, compared to the highly organized arrays of the desmosomes [25].

A number of questions remain open, the most important being the identity of the proteins involved in OSER stabilization. We have ruled out the obvious candidates, represented by the coiled-coil domain-containing Climp63, and the LINC complex proteins that have been proposed to perform a similar role in the NE. This shows that, although the OSER membranes may originate from the NE at least in some cases [10,12], the molecular machinery involved in OSER stabilization is likely different from that specialized in NE maintenance. Because the ordered areas could be observed between both luminal and cytosolic ER membrane faces, it is possible that more than one protein component may be involved in ER stacking interactions. It is possible that proteins involved are not integral membrane proteins, as is the case with the members of GRASP family (e.g., GRASP65), which are peripherally inserted into the Golgi membranes and stack the Golgi cisterns upon oligomerization [44-47]. Also, it remains to be determined whether the formation of the OSER stacks is primed by the interactions between adjacent ER membranes via the ordered macromolecular complexes, or whether the protein crowding and array formation is preceded by the formation of a membrane stack, which is in turn brought about by an independent mechanism.

## Conclusion

Our results support a hypothesis according to which active maintenance of organized smooth ER (and, by extension, the regular ER sheets) may be achieved in part by molecular tethering complexes. The potential significance of our findings is underscored by the recently shown cell cycle dependent 'tug of war' between sheet and tubular ER morphologies [5]. It is obvious that the molecules involved in ER sheet stabilization and stacking, together with structural proteins that act to stabilize the tubular ER morphology, e.g. reticulons and DP1 [7], must play key roles in the ER remodelling. Identification of the ER sheet stabilizing and tethering proteins, called for by the findings presented here, would help us understand the relationship between the ER morphologies, the cell cycle and the various pathologies that affect them.

## Authors' contributions

VMK conceived and designed the study, performed fluorescence and electron microscopy and drafted the manuscript; BZ performed cryo-sectioning and electron microscopy, participated in the design of the study and helped draft the manuscript.

## References

[B1] Shibata Y, Voeltz GK, Rapoport TA (2006). Rough sheets and smooth tubules. Cell.

[B2] Voeltz GK, Rolls MM, Rapoport TA (2002). Structural organization of the endoplasmic reticulum. EMBO reports.

[B3] Baumann O, Walz B (2001). Endoplasmic reticulum of animal cells and its organization into structural and functional domains. Int Rev Cytol.

[B4] Voeltz GK, Prinz WA (2007). Sheets, ribbons and tubules - how organelles get their shape. Nature reviews.

[B5] Puhka M, Vihinen H, Joensuu M, Jokitalo E (2007). Endoplasmic reticulum remains continuous and undergoes sheet-to-tubule transformation during cell division in mammalian cells. The Journal of cell biology.

[B6] Hu J, Shibata Y, Voss C, Shemesh T, Li Z, Coughlin M, Kozlov MM, Rapoport TA, Prinz WA (2008). Membrane proteins of the endoplasmic reticulum induce high-curvature tubules. Science (New York, NY).

[B7] Voeltz GK, Prinz WA, Shibata Y, Rist JM, Rapoport TA (2006). A class of membrane proteins shaping the tubular endoplasmic reticulum. Cell.

[B8] Crisp M, Liu Q, Roux K, Rattner JB, Shanahan C, Burke B, Stahl PD, Hodzic D (2006). Coupling of the nucleus and cytoplasm: role of the LINC complex. The Journal of cell biology.

[B9] Klopfenstein DR, Klumperman J, Lustig A, Kammerer RA, Oorschot V, Hauri HP (2001). Subdomain-specific localization of CLIMP-63 (p63) in the endoplasmic reticulum is mediated by its luminal alpha-helical segment. The Journal of cell biology.

[B10] Snapp EL, Hegde RS, Francolini M, Lombardo F, Colombo S, Pedrazzini E, Borgese N, Lippincott-Schwartz J (2003). Formation of stacked ER cisternae by low affinity protein interactions. The Journal of cell biology.

[B11] Okiyoneda T, Harada K, Takeya M, Yamahira K, Wada I, Shuto T, Suico MA, Hashimoto Y, Kai H (2004). Delta F508 CFTR pool in the endoplasmic reticulum is increased by calnexin overexpression. Molecular biology of the cell.

[B12] Pathak RK, Luskey KL, Anderson RG (1986). Biogenesis of the crystalloid endoplasmic reticulum in UT-1 cells: evidence that newly formed endoplasmic reticulum emerges from the nuclear envelope. The Journal of cell biology.

[B13] Shibata Y, Voss C, Rist JM, Hu J, Rapoport TA, Prinz WA, Voeltz GK (2008). The reticulon and dp1/yop1p proteins form immobile oligomers in the tubular endoplasmic reticulum. The Journal of biological chemistry.

[B14] Almsherqi ZA, Kohlwein SD, Deng Y (2006). Cubic membranes: a legend beyond the Flatland* of cell membrane organization. The Journal of cell biology.

[B15] Korkhov VM, Milan-Lobo L, Zuber B, Farhan H, Schmid JA, Freissmuth M, Sitte HH (2008). Peptide-based interactions with calnexin target misassembled membrane proteins into endoplasmic reticulum-derived multilamellar bodies. Journal of molecular biology.

[B16] Fidzianska A, Rowinska-Marcinska K, Hausmanowa-Petrusewicz I (2004). Coexistence of X-linked recessive Emery-Dreifuss muscular dystrophy with inclusion body myositis-like morphology. Acta neuropathologica.

[B17] Gonzalez-Alegre P, Paulson HL (2004). Aberrant cellular behavior of mutant torsinA implicates nuclear envelope dysfunction in DYT1 dystonia. J Neurosci.

[B18] Parmley RT, Spicer SS, Garvin AJ (1976). Multilaminar endoplasmic reticulum and abnormal mitosis in Hodgkin tumor cells. Cancer research.

[B19] Bernales S, McDonald KL, Walter P (2006). Autophagy counterbalances endoplasmic reticulum expansion during the unfolded protein response. PLoS biology.

[B20] Korkhov VM (2009). GFP-LC3 labels organised smooth endoplasmic reticulum membranes independently of autophagy. J Cell Biochem.

[B21] Smith JM (1999). Ximdisp - A visualization tool to aid structure determination from electron microscope images. Journal of structural biology.

[B22] Crowther RA, Henderson R, Smith JM (1996). MRC image processing programs. Journal of structural biology.

[B23] Shaner NC, Campbell RE, Steinbach PA, Giepmans BN, Palmer AE, Tsien RY (2004). Improved monomeric red, orange and yellow fluorescent proteins derived from Discosoma sp. red fluorescent protein. Nature biotechnology.

[B24] Warren DT, Zhang Q, Weissberg PL, Shanahan CM (2005). Nesprins: intracellular scaffolds that maintain cell architecture and coordinate cell function?. Expert reviews in molecular medicine.

[B25] Al-Amoudi A, Diez DC, Betts MJ, Frangakis AS (2007). The molecular architecture of cadherins in native epidermal desmosomes. Nature.

[B26] Schrag JD, Bergeron JJ, Li Y, Borisova S, Hahn M, Thomas DY, Cygler M (2001). The Structure of calnexin, an ER chaperone involved in quality control of protein folding. Mol Cell.

[B27] Nehls S, Snapp EL, Cole NB, Zaal KJ, Kenworthy AK, Roberts TH, Ellenberg J, Presley JF, Siggia E, Lippincott-Schwartz J (2000). Dynamics and retention of misfolded proteins in native ER membranes. Nature cell biology.

[B28] Anderson RG, Orci L, Brown MS, Garcia-Segura LM, Goldstein JL (1983). Ultrastructural analysis of crystalloid endoplasmic reticulum in UT-1 cells and its disappearance in response to cholesterol. Journal of cell science.

[B29] Senda T, Yoshinaga-Hirabayashi T (1998). Intermembrane bridges within membrane organelles revealed by quick-freeze deep-etch electron microscopy. Anat Rec.

[B30] Boisrame A, Chasles M, Babour A, Beckerich JM, Gaillardin C (2002). Sbh1p, a subunit of the Sec61 translocon, interacts with the chaperone calnexin in the yeast Yarrowia lipolytica. Journal of cell science.

[B31] Vergeres G, Yen TS, Aggeler J, Lausier J, Waskell L (1993). A model system for studying membrane biogenesis. Overexpression of cytochrome b5 in yeast results in marked proliferation of the intracellular membrane. Journal of cell science.

[B32] Takei K, Mignery GA, Mugnaini E, Sudhof TC, De Camilli P (1994). Inositol 1,4,5-trisphosphate receptor causes formation of ER cisternal stacks in transfected fibroblasts and in cerebellar Purkinje cells. Neuron.

[B33] Ohkuma M, Park SM, Zimmer T, Menzel R, Vogel F, Schunck WH, Ohta A, Takagi M (1995). Proliferation of intracellular membrane structures upon homologous overproduction of cytochrome P-450 in Candida maltosa. Biochim Biophys Acta.

[B34] Yamamoto A, Masaki R, Tashiro Y (1996). Formation of crystalloid endoplasmic reticulum in COS cells upon overexpression of microsomal aldehyde dehydrogenase by cDNA transfection. Journal of cell science.

[B35] Bouchet-Marquis C, Zuber B, Glynn AM, Eltsov M, Grabenbauer M, Goldie KN, Thomas D, Frangakis AS, Dubochet J, Chretien D (2007). Visualization of cell microtubules in their native state. Biology of the cell/under the auspices of the European Cell Biology Organization.

[B36] Garvalov BK, Zuber B, Bouchet-Marquis C, Kudryashev M, Gruska M, Beck M, Leis A, Frischknecht F, Bradke F, Baumeister W (2006). Luminal particles within cellular microtubules. The Journal of cell biology.

[B37] Gruska M, Medalia O, Baumeister W, Leis A (2008). Electron tomography of vitreous sections from cultured mammalian cells. Journal of structural biology.

[B38] Zuber B, Chami M, Houssin C, Dubochet J, Griffiths G, Daffe M (2008). Direct visualization of the outer membrane of mycobacteria and corynebacteria in their native state. Journal of bacteriology.

[B39] Zuber B, Haenni M, Ribeiro T, Minnig K, Lopes F, Moreillon P, Dubochet J (2006). Granular layer in the periplasmic space of gram-positive bacteria and fine structures of Enterococcus gallinarum and Streptococcus gordonii septa revealed by cryo-electron microscopy of vitreous sections. Journal of bacteriology.

[B40] Bouchet-Marquis C, Starkuviene V, Grabenbauer M (2008). Golgi apparatus studied in vitreous sections. J Microsc.

[B41] Salje J, Zuber B, Lowe J (2009). Electron cryomicroscopy of E. coli reveals filament bundles involved in plasmid DNA segregation. Science (New York, NY).

[B42] Zuber B, Nikonenko I, Klauser P, Muller D, Dubochet J (2005). The mammalian central nervous synaptic cleft contains a high density of periodically organized complexes. Proceedings of the National Academy of Sciences of the United States of America.

[B43] Al-Amoudi A, Frangakis AS (2008). Structural studies on desmosomes. Biochemical Society transactions.

[B44] Barr FA, Nakamura N, Warren G (1998). Mapping the interaction between GRASP65 and GM130, components of a protein complex involved in the stacking of Golgi cisternae. The EMBO journal.

[B45] Barr FA, Puype M, Vandekerckhove J, Warren G (1997). GRASP65, a protein involved in the stacking of Golgi cisternae. Cell.

[B46] Shorter J, Watson R, Giannakou ME, Clarke M, Warren G, Barr FA (1999). GRASP55, a second mammalian GRASP protein involved in the stacking of Golgi cisternae in a cell-free system. The EMBO journal.

[B47] Wang Y, Satoh A, Warren G (2005). Mapping the functional domains of the Golgi stacking factor GRASP65. The Journal of biological chemistry.

